# Retraction: Immunoglobulin G Expression in Lung Cancer and Its Effects on Metastasis

**DOI:** 10.1371/journal.pone.0228444

**Published:** 2020-01-24

**Authors:** The PLOS ONE Editors

Following the publication of this article [1], concerns have been raised regarding Figure 10 and Figure 11:

- In Figure 10B, the top left corner of the Untreated Beas2B panel appears similar to the bottom right corner of the siRNA-IGHG1 treated Beas2B panel. The corresponding author indicates that this was the result of an error in figure preparation and offered a replacement image correcting all three Beas2B panels.
- In Figure 11, similarities have been detected between the right-hand side of the Untreated A549 0hr panel and the right-hand side of the siRNA-IGHG1 treated A549 0hr panel. The authors have not provided an explanation for this concern and noted that the underlying data to support Figure 11 are not available.

In light of the unresolved concern about Figure 11 that calls into question the integrity of the reported results, the *PLOS ONE* Editors retract this article.

TH agreed with the retraction. JG did not agree with the retraction. GH did not confirm agreement or disagreement with the retraction decision. CJ, YW, and XW did not respond.
